# Web Service for HIV Drug Resistance Prediction Based on Analysis of Amino Acid Substitutions in Main Drug Targets

**DOI:** 10.3390/v15112245

**Published:** 2023-11-11

**Authors:** Anastasiia Iu. Paremskaia, Anastassia V. Rudik, Dmitry A. Filimonov, Alexey A. Lagunin, Vladimir V. Poroikov, Olga A. Tarasova

**Affiliations:** 1 Department of Bioinformatics, Pirogov Russian National Research Medical University, Ostrovitianov Str. 1, Moscow 117997, Russia; alexey.lagunin@ibmc.msk.ru; 2 Live Sciences Research Center, Moscow Institute of Physics and Technology, National Research University, Institutsky Lane 9, Dolgoprudny 141700, Russia; 3 Laboratory of Structure-Function Based Drug Design, Institute of Biomedical Chemistry, 10 bldg. 8, Pogodinskaya Str., Moscow 119121, Russia; rudik_anastassia@mail.ru (A.V.R.); dmitry.filimomov@ibms.msk.ru (D.A.F.); vladimir.poroikov@ibmc.msk.ru (V.V.P.)

**Keywords:** HIV/AIDS, antiretrovirals, resistance, machine learning, random forest, supporting vector machines, web-service

## Abstract

Predicting viral drug resistance is a significant medical concern. The importance of this problem stimulates the continuous development of experimental and new computational approaches. The use of computational approaches allows researchers to increase therapy effectiveness and reduce the time and expenses involved when the prescribed antiretroviral therapy is ineffective in the treatment of infection caused by the human immunodeficiency virus type 1 (HIV-1). We propose two machine learning methods and the appropriate models for predicting HIV drug resistance related to amino acid substitutions in HIV targets: (i) k-mers utilizing the random forest and the support vector machine algorithms of the scikit-learn library, and (ii) multi-n-grams using the Bayesian approach implemented in MultiPASSR software. Both multi-n-grams and k-mers were computed based on the amino acid sequences of HIV enzymes: reverse transcriptase and protease. The performance of the models was estimated by five-fold cross-validation. The resulting classification models have a relatively high reliability (minimum accuracy for the drugs is 0.82, maximum: 0.94) and were used to create a web application, HVR (HIV drug Resistance), for the prediction of HIV drug resistance to protease inhibitors and nucleoside and non-nucleoside reverse transcriptase inhibitors based on the analysis of the amino acid sequences of the appropriate HIV proteins from clinical samples.

## 1. Introduction

One of the major challenges in the fight against HIV infection is the emergence of drug resistance, which poses a serious threat to public health. Several studies have shown an alarming increase in drug resistance to antiretroviral drugs, including acquired resistance, which complicates the effective treatment of the disease [1,2,3,4]. Indeed, the likelihood of developing resistance to at least one antiretroviral drug is high and can lead to treatment failure in about 65% of cases [5]. In particular, the development of resistance to both nucleoside and non-nucleoside reverse transcriptase inhibitors, the main classes of drugs used to treat HIV, now exceeds 50% [6].

According to UNAIDS, this problem represents a serious challenge to more than 38 million people living with HIV worldwide, including more than 1.7 million children under the age of fifteen. Moreover, the likelihood of primary resistance to non-nucleoside reverse transcriptase inhibitors (NNRTIs) is projected to result in a significant increase in mortality rates and new cases of HIV infections [7]. Since the development of HIV drug-resistance is often associated with incomplete suppression of virus replication [8], it can increase HIV infection progression, which, in turn, leads to the emergence of new viral strains that can be transmitted to other persons.

To increase the efficacy of antiretroviral therapy (ARVT) and to minimize the spread of drug-resistant HIV, it is crucial to conduct viral load testing. Measurement of viral load in a patient’s blood helps determine if their antiretroviral therapy is effective in suppressing replication. This plays an important role in ensuring treatment adherence and selecting the most effective combinations of antiretroviral drugs to minimize resistance. For various reasons some patients demonstrate high viral loads even when taking antiretroviral drugs due to development of HIV drug resistance [9,10,11]. Therefore, the development of methods for the prediction of HIV drug resistance is of high importance.

Experimental methods for estimating resistance include genotypical or phenotypical assays.

Phenotypic tests assess the replicative ability of the virus after exposure to specific antiretroviral drugs at different concentrations. Phenosense™ and Antivirogram™ are examples of phenotypic test systems for evaluating HIV drug resistance [12]. To conduct the analysis, it is necessary to have a plasma sample with a viral load of over 500 copies/mL [13]. The measure of virus susceptibility to various drugs is the fold ratio (FR) value, calculated as the ratio of the semi-effective inhibiting concentration of a specific drug (IC_50_) for a specific variant of HIV to the IC_50_ for a wild type of HIV. The FR is measured by comparing the IC_50_ for a sample of a virus isolated from a patient with the IC_50_ thresholds for a drug-sensitive reference virus (CNDO strain) containing the protease sequences and reverse transcriptase sequences of the NL4-3 HIV 1 strain [14].

In clinical practice, genotyping is the most commonly used method for detecting HIV drug resistance. Genotyping is based on sequencing HIV samples to identify amino acid substitutions responsible for conferring resistance to certain antiretroviral drugs. However, interpreting the results of genotypic tests and predicting the level of resistance has become an important bioinformatics issue, and various approaches have been developed to help clinicians tackle this issue. 

There are several databases containing HIV genotype data along with various associated data, including resistance, treatment, subtypes, etc., such as the HIV database developed in Los Alamos National Laboratory (LANL) [15], the resources of the EuResist Project [16], and the Stanford HIV Drug Resistance Database [17]. The data in LANL can be used for the analysis of the prevalence of viral polymorphisms associated with resistance to a particular drug [18], analysis of conserved and variable domains among HIV variants [19], and studying the diversity of viruses and more [20]. Various methods were developed for the automated subtyping of HIV sequences [21], predicting responses to antiretroviral therapy [22], and drug exposure [23]. Rule-based systems, machine learning algorithms, and deep learning systems have been developed to predict drug resistance [24,25,26,27,28,29,30,31,32].

The study of the genetic variants of HIV in patients taking antiretroviral therapy revealed variants that were associated and not associated with drug resistance. Current approaches for computational HIV drug resistance prediction include those that are based on various machine learning algorithms [27,33,34] such as artificial neural networks and deep learning [27], random forest [34,35], and support vector machines [36,37,38].

The study carried out by Qihang Cai with co-authors [27] aimed to develop machine learning regression models for 21 drugs based on amino acid sequences with fixed lengths (99 sequences of HIV protease, 240 sequences of HIV reverse transcriptase, and 288 sequences of HIV integrase) and support vector machines (SVM). They used a random forest algorithm to weight the SVM model with a radial basis function and incorporated three different weight estimation methods using weight information in the SVM approach. Amino acid residues were represented as a 7-dimensional vector describing the physicochemical properties relevant to drug-protein interactions. The study shows that the use of an SVM based on radial basis functions with RF weights can achieve high coefficients of determination ($R^{2}$) above 0.8 for 16 drugs.

Partial least squares, random forest, LightGBM, and support vector regression were used to develop predictive models using molecular field parameters as predictors [28]. Steric and electrostatic molecular field calculations were performed on 3D protease structures obtained using homological modelling. The general number of sequences in the training sets and the test sets was 3719 and 934, respectively. The accuracy of the classification models ranged from 0.89 to 0.92 for 8 drugs, and the coefficients of determination ($R^{2}$) varied from 0.5 to 0.86.

Chen-Hsiang Shen and co-authors used two machine learning algorithms, random forest, and k-nearest neighbor in the classification process [36]. The feature vector in this case is calculated by summing the distances between the Cα atoms along each arc of the Delaunay triangulation. The studied sets included from 11,314 to 13,795 unique sequences of HIV PR mutants and from 4540 to 259,347 sequences of RT mutants. The arc connects two amino acids of the given type. This allowed the prediction of protease and reverse transcriptase inhibitor resistance with an accuracy varying from 0.97 to 0.99. The accuracy varied depending on the drug.

The collection of sequence and resistance data, as well as the active development of computational algorithms, provide an opportunity to create web services focused on predicting HIV drug resistance through amino acid sequences. In particular, the HIV Drug Resistance Database includes a tool, the HIVdb program for predicting drug resistance based on penalty points and a statistical evaluation of reduced susceptibility [31]. The Geno2pheno_[resistance]_ tool provides the ability to predict HIV resistance based on genotype data using regression models [30] and has some additional models aimed at predicting coreceptor usage [39] and evaluating resistance based on NGS data [40]. SHIVA is another tool developed for this purpose [32]. It is a web service that offers access to models created using random forest and linear interpolation [32]. Given the importance of HIV drug resistance in clinical practice, particularly in low- or middle-income countries, new computational methods focusing on the prediction of HIV drug resistance are continually emerging.

In this study we used our approaches developed earlier for the representation of HIV sequences that demonstrated their applicability for drug resistance prediction [37] and text mining for named entities recognition [41], respectively: (1) k-mers and (2) multi-n-grams. The datasets for training machine learning algorithms were collected from the Stanford HIV Drug Resistance Database (StHIVdb). K-mers represent short sequences of corresponding peptides (6–13 amino acids) constructed from a whole sequence. They were used in our previous study for building models aimed at HIV resistance prediction and resulted in reasonable accuracy [37]. Multi-n-grams were initially developed for representing texts for the purposes of named entity recognition (NER) and their use in the Bayesian approach allowed us to achieve a reasonable accuracy of NER even for highly imbalanced datasets [41]. Here, we utilized these methods for amino acid sequence representation along with machine learning algorithms to create models with high accuracy in predicting HIV resistance and developed a web application. This tool provides a reliable phenotypic interpretation of virus variants of amino acid substitutions in HIV protease and reverse transcriptase isolated from patients, helping clinicians prescribe appropriate antiretroviral drug combinations with a lower probability of treatment failure due to drug resistance.

## 2. Materials and Methods

### 2.1. Data Processing

We used ‘genotype–phenotype’ datasets, which describe a relationship between a viral genotype and the phenotypic manifestation of resistance to protease, and reverse transcriptase and integrase inhibitors from the Stanford HIV Drug Resistance Database [27]. To build models for the web service, we used high quality filtered datasets available for downloading from StHIVdb. To maximize the applicability of the models built in our approach, all available subtypes and viral variants of HIV-1 were used to model resistance. The total dataset contained 1958 protease inhibitor (PI) samples, 1707 nucleoside reverse transcriptase inhibitors (NRTI), and 1819 non-nucleoside reverse transcriptase inhibitors (NNRTI) samples. The samples are the amino acid sequences of HIV enzymes with values representing the levels of phenotypic resistance. The experimental level of resistance provided in the dataset was determined using the Phenosense™ test system. The table structure for the three drug groups, PI, NRTI, and NNRTI, derived from StHIVdb consists of a column with unique sequence identifiers and several drug columns with the phenotypic test results. There are also columns with the HIV enzyme positions and their values for each sequence, followed by a complete list of the mutations present in the isolate. The single-letter amino acid code found in the sequences provides information about the amino acids that are changed in this virus subtype (in comparison with a wild-type variant), and there are mixtures—positions where the amino acid is not fully resolved during sequencing and there are two or three amino acids as possible interpretations. Some of the amino acid changes are represented by special symbols such as “#” (an insertion), “~” (a deletion), etc.

All possible combinations of amino acid residues in the position for the isolate were used to resolve the mixtures in the position. The different amino acid residues in the same position in the sequence were changed to each of the residues mentioned in the sequence of the specific sample. Considering that all possible combinations in the subtype sequence allow for obtaining the best predictive ability, based on the results of previous studies [34], this approach led to the appearance of identical sequences with different phenotypic assay results. To reduce noise, the FR values for duplicates were averaged. To develop machine learning models, the samples were divided into two classes, ‘sensitive’ and ‘resistant’, using the FR thresholds from StHIVdb [17] provided in Table 1. Resistance of all viral variants was determined for all drugs present in the dataset, which is the reason why the final number of samples differs for each drug. The number of samples included in the class of susceptible (S) and resistant (R) variants is shown in Table 1.

### 2.2. Representation of Amino Acid Sequences Based on k-mer and String Muti-n-Grams Descriptors

**Figure 1:**
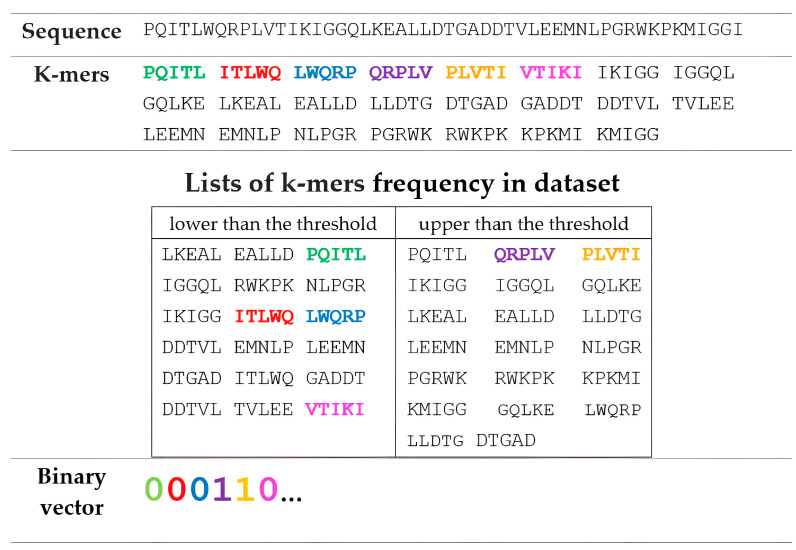
An example of generating a binary vector based on 5-mers for an amino acid sequence. The color of k-mers correspond to a particular digit in a binary vector.

We created classification models using a previously developed method that represented HIV sequences as binary vectors comprising ‘0’ and ‘1’ values indicating the occurrence of specific peptides (k-mers) in each amino acid sequence for a given drug [37]. K-mers are amino acid sequences of length k, which are corresponding sequences of the source protein. The study found that generated k-mers have a different frequency of occurrence in resistant and sensitive variants of HIV sequences. For each drug, we investigated the relationship between the accuracy of the model and the length of the k-mer. We used k-mers of length from 5 to 32 with an overlap of two amino acid residues. Such combinations of k-mer lengths and overlaps allowed us to achieve the best prediction accuracy. In the current work, we used a modified approach for building descriptors. In particular, we initially calculated the frequency of occurrence for each k-mer in a whole dataset for a particular drug. We set a frequency threshold of 0.1. If a specific k-mer was identified within the sequence at a frequency lower than the threshold, the vector was added with the value «0», otherwise the value «1» was added (Figure 1). Amino acid sequences were converted using the Python 3.8 programming language.

String descriptors implemented in the MultiPASS 2021 software for predicting biological activity based on the naïve Bayes approach have been applied to the reverse transcriptase and protease sequences of 240 and 99 amino acid residues, respectively. String descriptors (so-called multi-n-grams) are unique sequences of length from 1 to 15 generated from the sequences of HIV enzymes. To generate multi-n-grams, each symbol and their neighbors of level 1, 2, up to 15 [41] were used similarly to the generation of multilevel neighborhoods of atoms (MNA-descriptors) for chemical structures [41]. In this study we selected the optimal level of string descriptors for obtaining the highest prediction accuracy.

### 2.3. Model Development and Evaluation

The models were built using random forest and support vector machine machine learning methods from the library scikit learn 1.1.1.

Random forest is an ensemble learning technique that allows the combination of multiple decision trees to improve the accuracy and stability of the final prediction [42]. The algorithm creates a ‘forest’ of decision trees where each tree uses a randomly selected subset of the available features and training sets, making it more resistant to overfitting. During the prediction, the algorithm aggregates the results from all trees in the forest to produce the final output, with predictions based on majority votes from all trees.

Support vector machine provides the mapping of the input data from its original feature space into a high dimensional space where the data can be separated by a hyperplane [43]. The algorithm identifies the hyperplane that maximizes the margin between two classes of data points, with the margin being defined as the distance between the hyperplane and the nearest data points of both classes. The points closest to the hyperplane are called support vectors, and their positions determine the location of the hyperplane that separates the classes.

These algorithms were selected based on our previous study in which they demonstrate their effectiveness [37].

Hyperparameters were selected using a built-in function from the same library. For example, a random forest combination of three parameters was taken into account: function to measure the quality of separation, number of trees (from 81 to 201), and the maximum number of attributes used, which was either the square root of the total number of attributes or the base logarithm of two of the total number of attributes. The Gini index and Shannon entropy were considered as functions of the division quality assessment. Kernel function hyperparameters C and gamma were selected for the optimization of the support vector machine algorithm. Hyperparameter C determines the balance of bandwidth between classes and gap violations, i.e., the appearance of instances on the wrong side or in the middle of the band. The gamma hyperparameter is responsible for the individual input of each instance.

The MultiPASSR program is a specially developed version of MultiPASS 2021. It uses a Bayesian approach to predict resistance to the main anti-HIV drugs similarly to the prediction of the biological activity spectra for substances (the PASS program) [44]. MultiPASSR predicts the probability of samples belonging to the class of resistant samples (P_b_) and belonging to the class of susceptible—P_s_. The probabilities are estimated by analyzing the frequency of the occurrence of the multi-grams contained in a sequence in the sets of sequences corresponding to resistant and susceptible HIV variants.

The accuracy of the models was evaluated using a five-fold cross validation (5-fold CV), for which the data for each drug were randomly divided into 5 equal sets. Each dataset then served as a test set, and model training was provided for the remaining four sets. The models were evaluated using sensitivity, specificity, balanced accuracy, precision, and AUC metrics as averages on five control sets. The AUC (area under the curve of the receiver operating characteristic) is calculated by finding the area below the curve, which shows the relationship between sensitivity and specificity at different thresholds applied to the classification model. The AUC metric shows the overall quality of the classification, independent of the threshold. The value of the AUC metric can be in the range of 0 to 1, where the value of 0.5 corresponds to the random prediction of the class and the value of 1 corresponds to the ideal classifier. The AUC metric has a number of advantages because it: (1) does not depend on the threshold value of the model used to determine the class; and (2) is applicable to samples with a large number of features and non-linear dependencies as well as to unbalanced samples.

## 3. Results and Discussion

### 3.1. K-mer Based Model Evaluation

**Figure 2:**
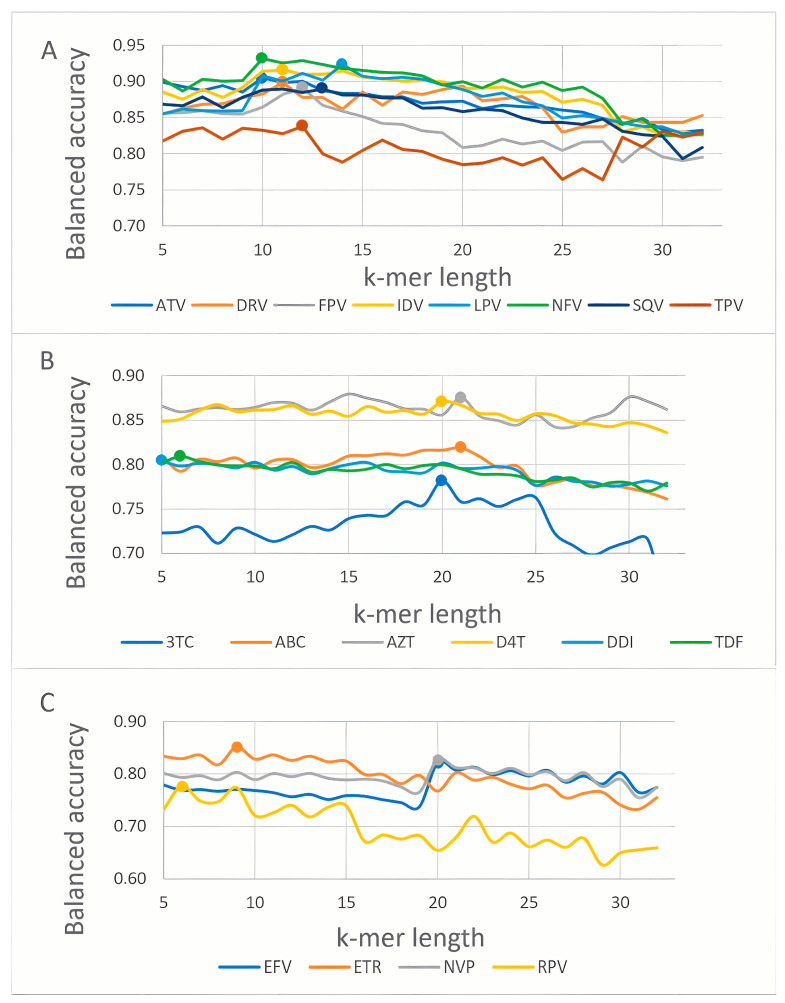
The relationship between a balanced accuracy and a length of encoded peptides for PI (**A**), NRTI (**B**) and NNRTI (**C**). Explanation of the abbreviations is provided in Table 2.

We estimated the k-mer lengths corresponding to the highest balanced prediction accuracy for most drugs of each class (PI, NRTI, NNRTI). Figure 2 demonstrates the relationship between the balanced prediction accuracy and various lengths of k-mers. K-mer length for protease inhibitors corresponding to the best models was between 10 and 14 amino acid residues (Figure 2A). The accuracy of the machine learning models was the highest at length k-mer 20 for lamivudine and stavudine, 21 for abacavir and zidovudine, lengths 5 and 6 showed maximum precision for didanosine and tenofovir, respectively (Figure 2B). According to the balanced accuracy curve (Figure 2C), it can be noted that the peaks for etravirine and rilpivirine were achieved using short k-mers, while for nevirapine and efavirenz, it was necessary to use longer sequences to achieve the highest accuracy.

By analyzing the relationship between the length of the k-mers and the accuracy of the models, one may conclude that there are some associations between the distribution of amino acid changes and resistance to a particular drug. The k-mers of a specific length serve to map the distribution, resulting in the highest value for model accuracy.

Based on the results of the evaluation of the models, final models using k-mer lengths corresponding to the highest balanced accuracy were selected. The average accuracy of the five control sets generated during 5-fold CV is presented in Table 2. The algorithm that demonstrated the best performance BA metric was selected for the web-based prediction of resistance to each drug.

The balanced accuracy of protease inhibitors varied from 0.84 to 0.93. The highest accuracy was achieved with nelfinavir, whereas tipranavir showed the lowest accuracy. Of the best eight models, two for fosamprenavir and tipranavir were constructed using the random forest algorithm, in the other cases the best result was achieved by the support vector machine.

The balanced accuracy for NRTI varied from 0.78 to 0.88. Efficient models were obtained by random forest for all drugs except lamivudine.

The best model for NNRTI was obtained for etravirine, with a balanced accuracy of 0.84 and an AUC of 0.94.

### 3.2. String Multi-n-Gram Descriptors for HIV-Resistance Prediction

The string descriptors for enzyme sequences were derived from MultiPASS. Table 3 presents the accuracy of models created using string descriptors and the naïve Bayes approach implemented in MultiPASSR using 5-fold CV.

We achieved a reasonable accuracy of HIV drug resistance prediction using the naïve Bayes algorithm with string multi-n-gram descriptors. The accuracy for most of drugs used was comparable to the levels achieved by k-mers and previously developed algorithms. In our earlier work [37], we demonstrated the effectiveness of the naïve Bayes algorithm for predicting HIV drug resistance using short amino acid sequences (k-mers). We also recently investigated the feasibility of using multi-n-grams for named entity recognition in scientific texts [41]. This work demonstrates the applicability of multi-n-grams and the naïve Bayes algorithm for building models to predict HIV drug resistance. It should be emphasized that multi-n-grams differ from k-mers because they include several substrings of different lengths up to a defined threshold (in this work, it is equal to 15) starting from each amino acid of the HIV enzyme. This study examines the applicability of an algorithm developed for named entity recognition in scientific texts, which has been modified for classifying HIV variants, i.e., drug resistance prediction. This observation demonstrates the applicability of certain algorithms that can handle highly imbalanced datasets to a variety of biological tasks.

When looking at the results of predicting resistance based on the developed models, it is possible to make some observations. More specifically, the balanced accuracy generally increases for k-mers lengths starting from 9 and gradually decreases beyond length 15. This length is probably sufficient to recognize substitutions that are located closely to each other. Hence, the V82F/I84V [45] replacements have a correlation with the stability of all currently approved IPs. Replacement L76V is associated with resistance to DRV, FPV, and IDV. Moreover, its combination with M46I, I54V, V82A, and L90M significantly augments resistance to LPV [46].

An accuracy of 0.88 for NRTIs was achieved with the best model developed for zidovudine. Zidovudine, the first approved NTRI, continues to be used in combination with other antiretroviral drugs for HIV treatment according to Dubois et al. [47]. The drug is well-studied and used in pediatric and pregnant patients. It is well-known that a high accuracy rate is associated with the quality of the training dataset.

Regarding NNRTI prediction based on k-mer and string multi-n-grams descriptors for reliable prediction, the descriptors for etravirine and rilpivirine were sufficient even with both short k-mers and low thresholds of k-mers. However, for efavirenz and nevirapine in both cases, the descriptors described a long sequence length. The observations may be partially explained by the chemical structures of the inhibitors. Etravirine, an NNRTI, is a second generation treatment for HIV-1 infection. It is a potent inhibitor that maintains activity against wild HIV types and is the most resistant among NNRTI subtypes of HIV [48]. The model could be created with high accuracy due to the absence of noise in a small amount of data.

Table 4 presents a comparison of the our results and those from previous studies on building classification models, conducted by Rhee et al. [49] and Heider et al. [50]. For comparison, only accuracy metrics were used as balanced accuracy could not be obtained from the source for all drugs.

To estimate the performance of our approach using external test sets, first, we divided the randomized data set into two parts in a 2:1 ratio, where two parts were used as training and one part was used as an external test. For comparison purposes, we constructed training and test sets as follows: the training set included the sequences from the Stanford HIV Drug Resistance Database that came from studies published no later than in 2018, while the rest of the sequences (which came from the studies published after 2018) were used as the test set. Here, 2018 was used as a threshold because the web-services used for comparison were reported in scientific publications before 2018 and therefore some recently collected sequences might be new for those models. The resulting accuracies of prediction are provided in Table 4 separated by a slash (“/”) symbol after the original accuracy provided by the authors of the corresponding studies. In case we were unable to upload the test dataset to the web server due to technical limitations, we only included the accuracy reported by the authors of the cited studies in Table 4.

We would like to emphasize that although we provide the prediction accuracy using specially constructed training and test sets, these results may be biased because we do not know, which training sets were used for building the models for the freely-available web-services and whether the test sets are truly external for these web-services. Nevertheless, we hope that even such a comparison can be used for determining the place of our approach among others.

The quality of the models is high for protease inhibitors. The lower accuracy for nucleoside and non-nucleoside inhibitors may be due to both the noisy training data and the sequence processing method, as well as the use of an unfiltered dataset from the Stanford HIV Resistance Database in the test set (for sequences published after 2018), whereas a high-quality filtered dataset was used for training. Although the models for nucleoside and non-nucleoside reverse transcriptase inhibitors have lower accuracy than expected when the test set of sequences published after 2018 was used, they are still reliable for making predictions based on the results of validation using the division of the high quality data set into training and test sets.

### 3.3. WEB-Application

We developed a freely available web application on the Way2drug Platform [52], based on the constructed models.

**Figure 3:**
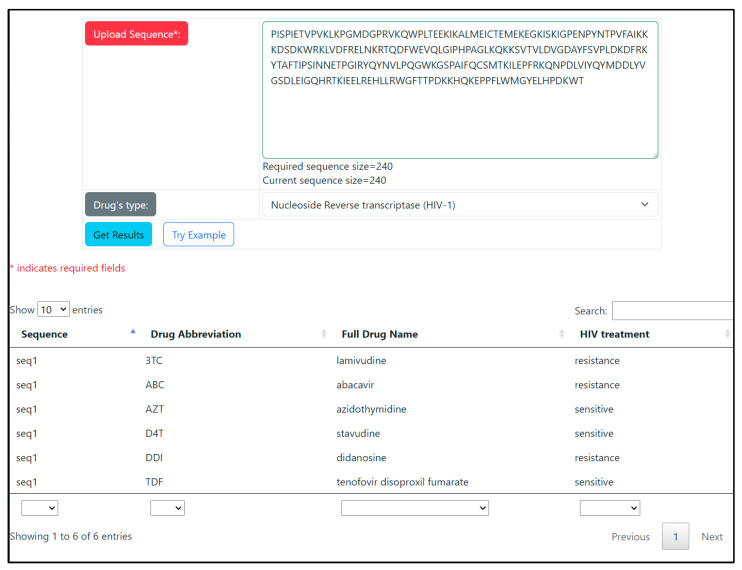
An example of the results for predicting resistance to antiretroviral drugs based on the amino acid sequence of reverse transcriptase.

The web application takes the amino acid sequences of HIV-1 reverse transcriptase or protease as an input (Figure 3). The models with the highest prediction performance for each drug were used for prediction. An additional option for predicting results is based on a consensus response of the models developed using multi-n-grams and k-mers through the logical operator ‘AND’.

To improve the results of drug resistance prediction using the web application, the alignment procedure to a reference sequence is carried out before the predictions. Alignment is carried out utilizing a Smith–Waterman algorithm modified for this purpose [53]. This feature allows users to input the amino acid sequences of protease and reverse transcriptase not limited by the length of sequences used for building the models. The amino acid sequences of 99 residues for HIV protease and 240 residues for reverse transcriptase were used for model training. Longer sequences are shortened to the necessary number of amino acids, considering the implemented local alignment. If the input sequence is shorter than the declared length, it undergoes local alignment to the reference sequence of HIV enzymes, and missing symbols are considered as deletions. There is some probability that long sequences of deletions can provide information about possible sites responsible for drug resistance. Thus, caution is advised when interpreting the accuracy of the prediction in such cases, ideally taking into account additional data.

The sequences undergo processing according to the aforementioned algorithm, which is based on k-mer frequency. The sequences are normalized, checked for length, and then divided into k-mers, with an overlap consisting of two amino acid residues. The length of the k-mers was chosen empirically for each drug based on the quality of prediction obtained for k-mer lengths ranging from five to thirty-two. To make predictions using MultiPASSR, string descriptors are generated for each sequence. This leads to a classification probability that indicates whether the sequence is ‘sensitive’ or ‘resistant’ to ARV preparations and the researcher can interpret the results of predictions.

## 4. Conclusions

The development and maintenance of web services for various biological and medical tasks are essential for improving convenience of use for scientists and clinicians in processing information. The represented tool can be used by researchers in drug development and to improve the effectiveness of therapy for people with laboratory confirmed HIV infection.

## Figures and Tables

**Table 1:** Balance of samples obtained for each anti-HIV drug.

Group	Drug	FR Threshold	Susceptible (S)	Resistant (R)	R/S ^1^
PI	Fosamprenavir (FPV)	4	1295	891	0.69
	Atazanavir (ATV)	3	804	649	0.81
	Indinavir (IDV)	3	1231	1003	0.81
	Lopinavir (LPV)	9	1069	853	0.80
	Nelfinavir (NFV)	3	1422	874	0.62
	Saquinavir (SQV)	3	1297	960	0.74
	Tipranavir (TPV)	2	731	396	0.54
	Darunavir (DRV)	10	697	130	0.19
NRTI	Lamivudine (3TC)	3	624	1417	2.27
	Abacavir (ABC)	3	806	1156	1.43
	Zidovudine (AZT)	3	1068	1014	0.94
	Stavudine (D4T)	1.5	1095	1000	0.91
	Didanosine (DDI)	1.5	947	1148	1.21
	Tenofovir (TDF)	1.5	1164	544	0.46
NNRTI	Efavirenz (EFV)	3	1072	995	0.93
	Nevirapine (NVP)	3	874	1181	1.35
	Etravirine (ETR)	3	382	172	0.45
	Rilpivirine (RPV)	3	121	88	0.73

^1^ R—resistant, S—susceptible, R/S represent ratio of dataset imbalance.

**Table 2:** Evaluation of the classification metrics for the k-mer based model.

Drug	k-mer Length	BA	Se	Sp	Pr	AUC	Method
Protease inhibitors							
Fosamprenavir (FPV)	12	0.89	0.88	0.90	0.86	0.95	RF
Atazanavir (ATV)	10	0.90	0.93	0.88	0.90	0.96	SVM
Indinavir (IDV)	11	0.92	0.92	0.91	0.91	0.96	SVM
Lopinavir (LPV)	14	0.91	0.92	0.91	0.89	0.96	SVM
Nelfinavir (NFV)	10	0.93	0.95	0.91	0.94	0.97	SVM
Saquinavir (SQV)	13	0.89	0.89	0.89	0.85	0.96	SVM
Tipranavir (TPV)	12	0.84	0.78	0.89	0.81	0.92	RF
Darunavir (DRV)	11	0.89	0.82	0.96	0.81	0.96	SVM
Nucleoside reverse transcriptase inhibitors							
Lamivudine (3TC)	20	0.78	0.88	0.69	0.87	0.86	SVM
Abacavir (ABC)	21	0.82	0.88	0.76	0.84	0.90	RF
Zidovudine (AZT)	21	0.88	0.90	0.86	0.86	0.95	RF
Stavudine (D4T)	20	0.87	0.88	0.86	0.85	0.94	RF
Didanosine (DDI)	5	0.80	0.83	0.77	0.81	0.88	RF
Tenofovir (TDF)	6	0.81	0.70	0.92	0.80	0.90	RF
Non-nucleoside reverse transcriptase inhibitors							
Efavirenz (EFV)	20	0.82	0.77	0.87	0.82	0.88	SVM
Nevirapine (NVP)	20	0.82	0.82	0.83	0.84	0.89	SVM
Etravirine (ETR)	9	0.84	0.74	0.95	0.87	0.94	SVM
Rilpivirine (RPV)	6	0.75	0.66	0.83	0.77	0.85	RF

BA—balanced accuracy, Se—sensitivity, Sp—specificity, Pr—precision, AUC—area under the curve of the receiver operating characteristic.

**Table 3:** Evaluation of models based on string multi-n-gram descriptors.

Drug	String Length	BA	Se	Sp	Pr	AUC
Protease inhibitors						
Fosamprenavir (FPV)	15	0.89	0.89	0.89	0.82	0.96
Atazanavir (ATV)	15	0.83	0.83	0.83	0.70	0.91
Indinavir (IDV)	15	0.90	0.90	0.90	0.91	0.96
Lopinavir (LPV)	15	0.91	0.91	0.91	0.83	0.97
Nelfinavir (NFV)	15	0.92	0.92	0.92	0.94	0.97
Saquinavir (SQV)	15	0.90	0.90	0.90	0.86	0.96
Tipranavir (TPV)	15	0.85	0.85	0.85	0.50	0.92
Darunavir (DRV)	6	0.89	0.89	0.89	0.35	0.96
Nucleoside reverse transcriptase inhibitors						
Lamivudine (3TC)	15	0.82	0.82	0.82	0.86	0.90
Abacavir (ABC)	15	0.86	0.86	0.86	0.89	0.93
Zidovudine (AZT)	15	0.88	0.89	0.88	0.87	0.95
Stavudine (D4T)	15	0.87	0.87	0.87	0.86	0.95
Didanosine (DDI)	15	0.82	0.82	0.82	0.81	0.90
Tenofovir (TDF)	15	0.80	0.80	0.80	0.53	0.89
Non-nucleoside reverse transcriptase inhibitors						
Efavirenz (EFV)	15	0.83	0.83	0.83	0.82	0.92
Nevirapine (NVP)	15	0.84	0.84	0.84	0.87	0.91
Etravirine (ETR)	6	0.84	0.84	0.84	0.34	0.90
Rilpivirine (RPV)	4	0.76	0.75	0.76	0.73	0.82

BA—balanced accuracy, Se—sensitivity, Sp—specificity, Pr—precision, AUC—area under the curve of receiver operating characteristic.

**Table 4:** Comparison of model accuracy with previous studies aimed at developing predictive web services.

Drug	Accuracy Rhee et al. [49]	Accuracy Heider et al. [50].	Accuracy Beerenwinkel et al. [51]	k-mer Approach	MultiPassR Result
	Protease inhibitors				
Fosamprenavir (FPV)	-/-	-/-	-/-	0.87/-	0.82/-
Atazanavir (ATV)	0.77 */0.76 **	0.88 */- **	- */0.94 **	0.90 ***/0.89 **	0.80 ***/0.74 **
Indinavir (IDV)	0.79/-	0.93/-	-/0.96	0.93/0.91	0.87/0.85
Lopinavir (LPV)	0.81/0.79	0.92/-	-/0.95	0.92/0.87	0.87/0.82
Nelfinavir (NFV)	0.82/-	0.91/-	-/0.93	0.92/0.90	0.90/0.87
Saquinavir (SQV)	0.84/-	0.89/-	-/0.92	0.88/0.81	0.87/0.78
Tipranavir (TPV)	-/	-/-	-/0.86	0.85/0.82	0.81/0.78
Darunavir (DRV)	-/0.79	-/-	-/0.90	0.92/0.86	0.88/0.84
	Nucleoside reverse transcriptase inhibitors				
Lamivudine (3TC)	0.90/0.97	0.90/-	-/0.95	0.82/0.79	0.81/0.80
Abacavir (ABC)	0.77/0.90	0.88/-	-/0.95	0.82/0.76	0.84/0.83
Zidovudine (AZT)	0.76/0.74	0.84/-	-/0.87	0.87/0.78	0.87/0.86
Stavudine (D4T)	0.78/0.78	0.84/-	-/0.89	0.87/0.79	0.85/0.87
Didanosine (DDI)	0.75/0.87	0.79/-	-/0.77	0.81/0.76	0.79/0.78
Tenofovir (TDF)	0.73/0.70	0.79/-	-/0.76	0.84/0.76	0.80/0.76
	Non-nucleoside reverse transcriptase inhibitors				
Efavirenz (EFV)	0.87/0.85	0.88/-	-/0.87	0.80/0.75	0.81/0.83
Nevirapine (NVP)	0.91/0.84	0.87/-	-/0.89	0.79/0.77	0.84/0.80
Etravirine (ETR)	-/0.82	-/-	-/-	0.84/0.78	0.84/0.77
Rilpivirine (RPV)	-/-	-/-	-/-	0.82/0.75	0.74/0.74

* The accuracy provided in the original study; only the accuracy of the qualitative classification is provided. ** The accuracy calculated using an external test set constructed using sequences published after the year 2018. If the accuracy was not calculated due to technical limitations or lack of data, “-” is provided. *** The accuracy calculated using an external test set constructed by dividing the randomized dataset into two parts in the ratio: 2:1, where two parts were used as training and one part was used as external test (performed only for our approach).

## Data Availability

The web service is freely available at http://way2drug.com/hiv-host/hvr (accessed on 8 November 2023). The datasets used for building models for the web-service is provided in Supplementary Materials.

## Supplementary Materials

The following supporting information can be downloaded at: https://www.mdpi.com/article/10.3390/v15112245/s1, Supplementary File S1: Datasets for each drug.
